# Pharyngeal Polysaccharide Deacetylases Affect Development in the Nematode *C. elegans* and Deacetylate Chitin *In Vitro*

**DOI:** 10.1371/journal.pone.0040426

**Published:** 2012-07-13

**Authors:** Ronald J. Heustis, Hong K. Ng, Kenneth J. Brand, Meredith C. Rogers, Linda T. Le, Charles A. Specht, Juliet A. Fuhrman

**Affiliations:** 1 Department of Biology, Tufts University, Medford, Massachusetts, United States of America; 2 Department of Medicine, University of Massachusetts, Worcester, Massachusetts, United States of America; New England Biolabs, United States of America

## Abstract

Chitin (β-1,4-linked-N-acetylglucosamine) provides structural integrity to the nematode eggshell and pharyngeal lining. Chitin is synthesized in nematodes, but not in plants and vertebrates, which are often hosts to parasitic roundworms; hence, the chitin metabolism pathway is considered a potential target for selective interventions. Polysaccharide deacetylases (PDAs), including those that convert chitin to chitosan, have been previously demonstrated in protists, fungi and insects. We show that genes encoding PDAs are distributed throughout the phylum *Nematoda*, with the two paralogs F48E3.8 and C54G7.3 found in *C. elegans*. We confirm that the genes are somatically expressed and show that RNAi knockdown of these genes retards *C. elegans* development. Additionally, we show that proteins from the nematode deacetylate chitin *in vitro*, we quantify the substrate available *in vivo* as targets of these enzymes, and we show that Eosin Y (which specifically stains chitosan in fungal cells walls) stains the *C. elegans* pharynx. Our results suggest that one function of PDAs in nematodes may be deacetylation of the chitinous pharyngeal lining.

## Introduction

Chitin, the homopolymer of β-1,4-linked-N-acetylglucosamine (GlcNAc), contributes to the mechanical strength and chemical impermeability of both the embryonic eggshell and the pharyngeal lining in nematodes. As such, this carbohydrate is of major structural importance during nematode development. Chitin is an insoluble, neutrally charged, chemically inert carbohydrate well-suited for its protective functions. It is synthesized in nematodes, but not in plants and vertebrates, many of which serve as hosts for parasitic roundworms. Understanding chitin metabolism will inform the development of interventions that selectively target nematodes important to agriculture and to the health of domesticated animals and humans.

Enzymes that function during major steps of chitin metabolism have been identified in a wide variety of species. Chitin synthases (which polymerize and deposit chitin) and chitinases (which hydrolyze chitin to its subunits) have previously been characterized in protists, fungi, and invertebrates including nematodes and insects. Recently, chitin deacetylases (which convert chitin to chitosan) have been identified in a range of organisms. Deacetylation has been demonstrated to be a versatile mechanism that influences cellular and organismal growth, as it converts chitin to a charged polymer that has increased solubility in aqueous environments and increased pliability. Chitin deacetylases alter the composition of the cyst wall in the protist *Entamoeba invadens* [1] and the cell wall of the fungus *Cryptococcus neoformans* [2], [3]. Chitin deacetylases also play essential roles during insect development [4], [5], [6], [7]. In *Drosophila melanogaster, serpentine* (*serp*) and *vermiform* (*verm*) genes encode chitin deacetylases that function in the chitin-lined embryonic tracheal tubes and that are essential for survival. This finding first established a role for chitin deacetylases in animals. Luschnig *et al.* (2006) noted a homolog of the *serp* and *verm* genes found in *C. elegans*: *Ce-lgx-1*/C54G7.3 bears both a predicted polysaccharide deacetylase (PDA) and predicted chitin-binding (CBD) domain; however, no functional work has established a role for chitin deacetylases in nematodes.

Chitin deacetylases, and all other PDAs, belong to the CAZY (http://www.cazy.org) carbohydrate esterase 4 family of enzymes. This family of enzymes acts on an array of different carbohydrates catalyzing the cleavage of ester linkages in xylan or amide linkages of GlcNAc in peptidoglycan and chitin. These enzymes function through a catalytic site termed the PDA domain, which is also referred to as the NodB homology domain. In *Rhizobium*, the NodB protein processes lipooligosaccharide signaling molecules. It serves the role of a polysaccharide deacetylase when it deacetylates chitooligosaccharides (short stretches of repeating GlcNAc monomers) during the production of these signaling molecules [8]. We used the protein sequences of this domain from previously characterized peptidoglycan and chitin deacetylases as query sequences in a comprehensive bioinformatic search to identify polysaccharide deacetylases among the *Nematoda* and by sequence similarity comparisons suggest that chitin is the substrate. In this paper, we show that in addition to the previously noted homolog of *serp* and *verm* (C54G7.3), a second PDA-encoding gene (F48E3.8) is found in *C. elegans*. We also demonstrate the presence of homologs in many other species of *Nematoda*, including important parasites of plants and vertebrates. We took advantage of *C. elegans* to investigate a role for this class of enzymes, building on existing publicly available data (NextDB, http://nematode.lab.nig.ac.jp) and published information [9], showing that the genes are expressed in the pharynx. We confirm that the genes are somatically expressed and present evidence that disruption of these genes results in a delay in developmental timing. Our results also include a demonstration that proteins derived from the nematode deacetylate chitin *in vitro*.

## Results

The first metazoan chitin deacetylase was identified as a novel peritrophic membrane protein from the cabbage looper *Trichoplusia ni* [10] and subsequent work has shown that insects have a large family of PDA-domain encoding genes, with nine homologs found in the red flour beetle *Tribolium castaneum* [7]. In *D. melanogaster*, *serp* and *verm* encode two chitin deacetylases which function during embryonic development [5], [6]. Luschnig *et al.* (2006) noted that these genes shared homology with the *C. elegans* gene *lgx-1*/C54G7.3 (hereafter referred to as C54G7.3), which bears a predicted chitin-binding domain (CBD) and a predicted polysaccharide deacetylase domain (PDA). Using the C54G7.3 predicted PDA protein sequence as a basis for bioinformatic analysis, we identified homologs in other nematodes, including important parasites of plants and animals and four free-living species from the genus *Caenorhabditis* (**Table 1**). In some nematode species, we identified a single predicted PDA sequence, but noted the presence of two homologs in all four *Caenorhabdita*. In *C. elegans*, F48E3.8 also encodes a putative polysaccharide deacetylase. Although we observed only a single homolog in the genome of the filarial parasite *B. malayi* (Bml_33340), using *SpPDA* as a search sequence against the Brugian endosymbiont *Wolbachia* returned the gene WolBm0147, from the TRS strain of this bacterium. As such, two PDAs may be expressed in *B. malayi*, one by the nematode and one by its endosymbiotic bacterium. In *Onchocerca volvulus*, a related filarial parasite, we identified a sequence apparently related to the *B. malayi* genomic sequence (contig33574) and one related to the *Wolbachia*-derived sequence (contig48143) based on an analysis of Sanger sequence repositories. PDAs appear highly conserved across the nematode phylum since at least one homologous sequence was found in species from Clades I, III, IVb and V. We were unable to identify homologs in other important vertebrate parasites: *Acanthocheilenema viteae*, *Brugia pahangi*, *Brugia timori*, *Dirofilaria immitis* and *Wuchereria bancrofti*. This may reflect a lack of available sequence data rather than the absence of genes or transcripts in these species. No human homolog was detected in our searches, although vertebrate homologs of glycosaminoglycan deacetylases (bearing protein sequence highly divergent from the NodB domain) have been identified [11].

**Table 1:** Nematode PDAs Identified Through Bioinformatics using resources from NCBI, Sanger, and www.nematode.net.

Species	Clade	NCBI Accession #	Previously Assigned Names or Retrieval Information Relevant for Other Databases	Total # of Residues in Archived Sequence	Residues Included in Alignments and Tree Relative to Archived Sequence
*Ancylostoma caninum*	V	N/A	Acan_isotig17215 (Nematode.net)	–	–
*Ascaris suum*	III	ADY42356^a^	ADY42356	–	–
		ADY43217^b^	ADY43217	735	578–733
*Brugia malayi*	III	AAW70738	WolBm0147, YP_197908	264	55–214
		EDP33092	Bml_33340; 14961.m05161 (TIGR)	1802	1469–1624
*Caenorhabditis brenneri*	V	EGT33229	CAEBREN_29772	2693	2373–2526
		EGT30475	CBN-LGX-1	666	321–481
*Caenorhabditis briggsae*	V	CAP34613	CBG_16715	2523	2202–2355
		CAP33224	CBG_14800	699	354–513
*Caenorhabditis elegans*	V	CCD67046	C54G7.3	1876	1500–1533, 1557–1650^c^
		ABX00803	F48E3.8	2444	2124–2277
*Caenorhabditis remanei*	V	EFO83068	CRE_00058	2545	2225–2378
		EFO82567	CRE_00232	682	335–498
*Loa loa*	III	EFO26644	LOAG_01839	416	83–237
*Meloidogyne incognita* d	IVb	AF531169	Msp9, MI00182 (Nematode.net),MI04101 (Nematode.net)	–	–
*Oesophagostomum dentatum*	V	N/A	Oden_isotig26807 (Nematode.net)	–	–
*Onchocerca flexuosa*	III	N/A	OF01019 (Nematode.net)	–	–
*Onchocerca volvulus*	III	N/A	contig33574 (Sanger)	–	–
		N/A	contig48143 (Sanger)	–	–
*Ostertagia ostertagi*	V	N/A	OS02548 (Nematode.net)	–	–
*Teladorsagia circumtincta*	V	N/A	Tcir_isotig26540 (Nematode.net)	–	–
*Trichinella spiralis*	I	EFV55667	Tsp_03980	1524	1190–1343

a,b The sequences ADY42356 and ADY43217 share 99% sequence identity within the predicted NodB homology domain, but only 32% sequence identity outside this region. ADY42356 has a truncated portion of the catalytic domain. We are unable to discern these as 1 or 2 homologs, but given the catalytic domain identity we use only ADY43217 for alignments.

c Based on our sequencing of this region of the gene we have shown that predicted residues 1534–1556 (relative to predicted sequence associated with Accession No. CCD67046) would not be present in the protein sequence.

d *M. incognita* Msp9 is a potential homolog which has sequence verification from two clones available from www.nematode.net. Msp30 (AY142120) shows some homology as well, but there is less supporting data.

**Figure 1:**
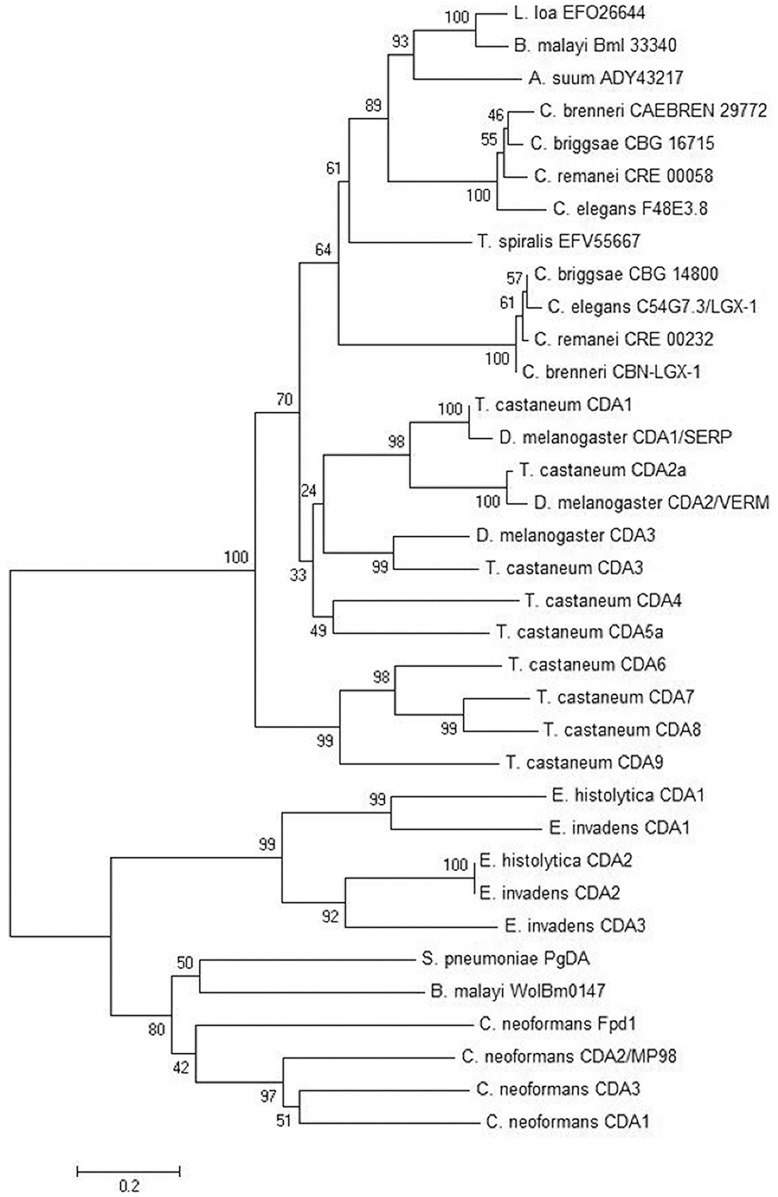
Phylogenetic tree showing the relationship of the putative *C. elegans* PDAs to previously characterized peptidoglycan or chitin deacetylases from bacteria, fungi and insects. A ClustalW alignment of residues predicted to encode the polysaccharide deacetylase domain of each protein was used to generate a phylogenetic tree by applying the minimum evolution method using the tree-drawing software Mega 4.0 (Kumar *et al.*, 2004). A bootstrap consensus tree based on 5000 replicates is depicted and bootstrapping values are shown. C54G7.3 and F48E3.8 are two predicted homologs in *C. elegans* and are related to distinct homologs in three other species from the genus *Caenorhabditis.* Other sequences are from the filarial parasitic nematodes *Brugia malayi* and *Loa loa* and from the non-filarial parasitic nematodes *Ascaris suum* and *Trichinella spiralis*. A homologous sequence derived from the *B. malayi* bacterial endosymbiont *Wolbachia sp.* TRS (WolBm0147) is also included along with sequences from the bacterium *Streptococcus pneumoniae*, the protists *Entamoeba histolytica* and *Entamoeba invadens*, the fungus *Cryptococcus neoformans*, and the insects *Drosophila melanogaster* and *Tribolium castaneum.*

We created a phylogenetic tree based on ClustalW alignment of the PDA catalytic domains (**Figure 1**). We used a subset of our newly identified nematode sequences and included only those predicted protein sequences generated from reliable transcript sequence data. In *C. elegans*, the exon-intron boundaries covering the predicted PDA-encoding domain of F48E3.8 were previously verified in ESTs; however, exon-intron boundaries spanning the predicted C54G7.3 PDA-encoding domain have not been verified (Wormbase WS229). We sequenced a region of C54G7.3 and found that the predicted 69-bp exon 28 was not expressed. We have therefore omitted 23 amino acids (SFKFKIKNFKKVIPNTLSLKNTI) from the protein associated with the NCBI Accession No. CCD67046. All our alignments and the phylogenetic tree reflect this correction.

The phylogenetic tree, generated using the minimum evolution (ME) method, shows that the nematode sequences are more closely related to each other than to the PDA sequences from bacteria, protists, fungi and insects (**Figure 1**). The sequence WolBm0147 derived from the endosymbiont of *B. malayi*, however, is most closely related to the only other bacterial sequence, *S. pneumoniae* PgDA (peptidoglycan deacetylase) included in our analysis. Our results replicate the branching pattern previously described for insect PDA proteins where the nine *T. castaneum* polysaccharide deacetylases were categorized into five groups. This organization was based on homology within the catalytic domain and domain architecture within the full primary sequence [7]. (In insects, all members of this family of proteins have been named chitin deacetylases although functional work has not specifically elucidated chitinous targets for some of these enzymes.) In *T. castaneum*, Group 1 and Group 2 CDA proteins have a chitin-binding domain (CBD), a low-density lipoprotein receptor domain (LDLa) and a chitin deacetylase domain (CDA). Group 3 and Group 4 CDA proteins have a CBD and a CDA domain (but no LDLa) and Group 5 CDA proteins have a CDA domain (but neither of the other 2 domains). Our phylogeny suggests that nematode PDAs are more closely related to the Groups 1–4 insect CDAs (*Tc*CDA1-5), than they are to the Group 5 CDAs (*Tc*CDA6-9). PFam analysis predicts the presence of a weak-scoring CBD in *C. elegans* F48E3.8 and C54G7.3, but not LDLa domains in these nematode sequences.

The relationship established here among *C. elegans* F48E3.8, its orthologs *C. brenneri* CAEBREN_29772, *C. briggsae* CBG_16715, and *C. remanei* CRE_00058, and the single genes detected from *A. suum*, *B. malayi*, *L. loa* and *T. spiralis*, reflects the same evolutionary profile described for these species when phylogenetic analysis is based on the ribosomal DNA sequences [12]. The filarial sequences are most closely related, share a most recent common ancestor with *A. suum*, an earlier common ancestor with the *Caenorhabdita*, and an even earlier common ancestor with *T. spiralis* (**Figure 1**). This is similar to the relationship among these species in the Blaxter *et al.* (1998) analysis. The distance between F48E3.8 orthologs and C54G7.3 orthologs suggests that significant variation occurred within the sequence of one or both genes following a likely gene duplication event that created paralogs in a common ancestor of the *Caenorhabdita*.

**Figure 2:**
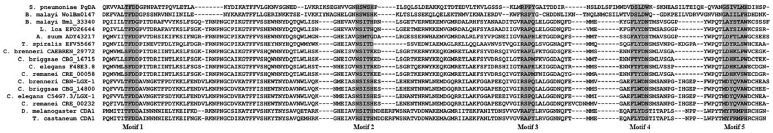
Alignment of protein sequences spanning the polysaccharide deacetylase domain (Nod B homology domain) for some newly identified nematode polysaccharide deacetylases and representative sequences from bacteria and insects. The catalytic domain of this family of enzymes has been previously characterized by the inclusion of five motifs essential for activity. Motifs 1 and 2 contribute residues necessary for co-factor binding; Motifs 1, 3, 4 and 5 contribute residues essential for acetyltransferase activity [13], [14], [15]. All five motifs are conserved in nematode PDAs. Some residues are highly conserved among nematodes but differ from the canonical residues observed in bacterial and/or insect sequences. In general, nematode sequences are more similar to the other metazoan homologs from insects – with large stretches of identity observed among nematode and insect sequences even outside the motifs. Our identified sequence WolBm0147, derived from *Wolbachia sp*. TRS, is more similar to the representative bacterial sequence from *Streptococcus pneumoniae* supporting a bacterial origin of this sequence.

The PDA domain has been designated as the NodB homology domain in the PFam database (NCBI Accession No. PF01522), and defines a catalytic site including five functional motifs that were first characterized in bacteria [13], [14] and later in fungi [15]. These motifs are also conserved in the insect CDA proteins [7]. Based on the alignment of the NodB domains from the newly identified nematode PDA proteins included in our tree, these five motifs are also conserved in predicted nematode PDA proteins (**Figure 2**). In bacterial and fungal sequences, specific residues in Motifs 1 and 2 have been assigned roles in binding to metal co-factors while specific residues in Motifs 1, 3, 4 and 5 have been shown to function as part of the catalytic acetyltransferase activity. The presence of the five motifs in all nematode-derived sequences suggests that they retain the ability to act on carbohydrates to deacetylate them.

Motif 1 has been identified as a TFDD sequence in other organisms and is highly conserved in nematodes as a (T/S)FDD sequence, where a threonine-to-serine substitution characterizes *Ce*C54G7.3 and its orthologs. In nematodes, motif 2 is an NSI(T/S)X. This is a notable exception to other organisms where the first and last residues of motif 2 have been reported as two histidine residues as they are in *S. pneumoniae* peptidoglycan deacetylase and the *Wolbachia* sequence WolBm0147. In the *Caenorhabdita*, NSISH is the fully conserved Motif 2 sequence. The nematode motif 3 sequence is an R(S/A)PX: the fourth residue is a variable amino acid which replaces the tyrosine residue found in that position for bacterial and insect sequences. Motif 4 in nematodes is an FXXDN and Motif 5 is a more variable, though not exceptionally divergent sequence, with a fully conserved tryptophan residue replacing the histidine found in bacterial and insect sequences at position 7. Among *Ce*C54G7.3 and its orthologs in the *Caenorhabdita*, the residues in Motifs 3–5 are fully conserved just as they are in Motifs 1 and 2. This alignment also underscores the similarity between our identified WolBm0147 sequence and that of the other representative bacterial sequence.

Functions for these newly classified nematode PDAs have not been previously established. *Ce*F48E3.8 and *Ce*C54G7.3 have been shown to be upregulated in the embryonic pharynx and both genes have multiple TRTTKRY binding sites for the PHA-4 transcription factor within their upstream regulatory regions [9]. The Nematode Expression Database (NEXTDB, http://nematode.lab.nig.ac.jp/) provides *in situ* hybridization data confirming that both genes are expressed in the developing embryonic pharynx. F48E3.8 (cosmid F48E3, clone 272e1 in the database) is detected in embryos, but not in other stages. C54G7.3 (cosmid C54G7, clones 325b6 and 208c10) is abundantly expressed in the pharynx of embryos, larvae and adults, with a spatiotemporal expression pattern similar to that of the pharyngeal chitin synthase *chs-2* (cosmid F48A11, clone 316g4), although *chs-2* is also detected in non-pharyngeal tissue (NEXTDB, http://nematode.lab.nig.ac.jp/). The expression of F48E3.8 and C54G7.3 in the *C. elegans* pharynx suggested that one possible target of these polysaccharide deacetylases could be the chitinous lining of the pharyngeal tract. In *D. melanogaster*, the two chitin deacetylases Serp and Verm target the chitin lining the embryonic trachea and act to regulate longitudinal (and possibly radial) growth of these epithelial tubes. This regulation is crucial for permitting efficient gas diffusion for the developing embryo [5], [6]. This mechanism of regulating epithelial tube growth may be applicable to other organisms and we reasoned that nematode homologs may affect development through their action on pharyngeal chitin.

**Figure 3:**
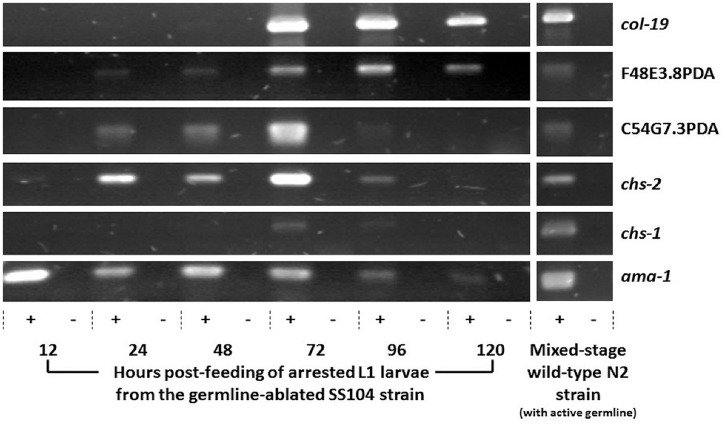
Developmental time course of F48E3.8 and C54G7.3 gene expression in germline-ablated *C. elegans* using RT-PCR. Synchronous non-permissive (germline-ablated) worms of this strain were grown on solid media and used to collect RNA from various time-points following L1 arrest at hatching and continuing into adulthood. First strand cDNA (+) was generated from each RNA extract (including No RT controls, –) and used as a template for PCR to analyze expression of the genes F48E3.8, C54G7.3, the eggshell chitin synthase *chs-1* and the pharyngeal chitin synthase *chs-2*. The gene *ama-1*, encoding the large subunit of RNA polII, was used as a positive control (as done by Johnstone and Barry 1996 and Veronico *et. al.* 2001) although we note that expression of the gene is not consistent as previously described. Ablation of the germline was confirmed by the attenuation in expression of *chs-1*. The transition to adult stages was confirmed by the expression of *col-19*, an adult specific collagen gene. All primers and PCR conditions are listed in Table S2.

Using semi-quantitative RT-PCR, we show that both genes are somatically expressed as they were detected in the SS104 strain grown under conditions that restrict the production of a germline (**Figure 3**). In our time course, both genes were first detected at 24 h following hatching, when grown at 25°C. Both PDA transcripts continued to be expressed at 48 h and 72 h. Adults first appeared at the 72 h time point, marked by the onset of expression of the adult specific collagen *col-19* [16] and consistent with our cytological observations (as approximately 75% of worms on the plates had an identifiable vulva at 72 h while none had this at 48 h). F48E3.8 and C54G7.3 genes showed clear expression despite the significant attenuation of the germline, evidenced by the trace expression of the eggshell chitin synthase gene *chs-1*. *chs-1* is abundantly expressed in the germline and can be easily detected by RT-PCR in similar time courses from wild-type worms [17]. In contrast to *chs-1*, the pharyngeal chitin synthase *chs-2* was previously shown to be expressed at early stages of development, to appear in the pharynx prior to the L1/L2 molt when assayed using GFP reporters, and to decline following the molt to adulthood (72 h) [17], [18], [19]. The expression patterns for the two PDAs were similar to *chs-2* from 24 h to 72 h in our experiment (**Figure 3**). However, C54G7.3 and *chs-2* expression declined following onset of adulthood (72 h), while F48E3.8 expression persisted through to 120 h as did *col-19* expression. Both PDAs, then, were expressed in the absence of a germline, but F48E3.8 appeared to persist into adulthood while C54G7.3 expression declined once all the worms reached adulthood. All three predicted transcripts of F48E3.8, but only one of the two predicted transcripts of C54G7.3, include sequence coding for the PDA domain. Importantly, our RT-PCR experiments were designed to detect all transcripts predicted to encode PDA domains; thus, our results show that transcripts capable of producing active deacetylase enzymes are expressed in the *C. elegans* soma. The demonstration of F48E3.8 expression in larval and adult stages contrasts with *in situ* data that showed expression was restricted to embryos (NEXTDB, http://nematode.lab.nig.ac.jp/). This may reflect increased sensitivity afforded by RT-PCR or differences in expression of alternative transcripts of F48E3.8.

**Figure 4:**
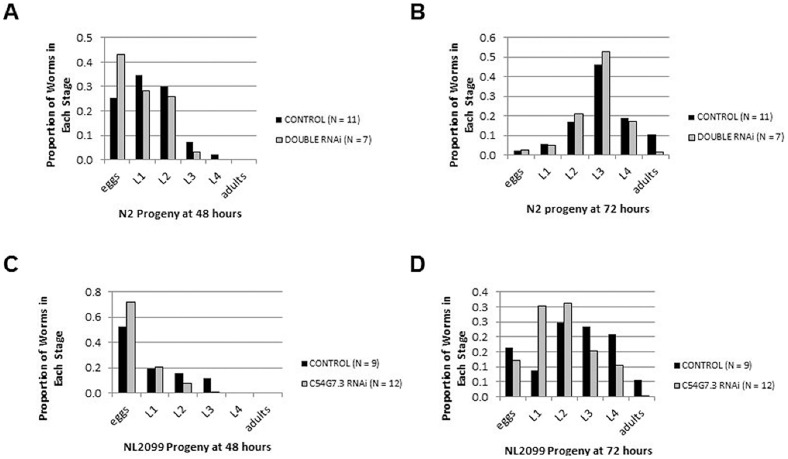
RNAi targeting chitin deacetylases affect developmental timing. dsRNA targeting both putative polysaccharide deacetylases F48E3.8 and C54G7.3 was introduced to the wild-type N2 strain (A,B) by feeding. dsRNA targeting the C54G7.3 gene alone was introduced to the RNAi-hypersensitive strain NL2099 (C,D) by feeding. Single L3 larvae were allowed to proceed through development while feeding before progeny were scored at 48 hours (A,C) and 72 hours (B,D). RNAi targeting both genes results in a delay in wild-type worm development that can also be observed when C54G7.3 is targeted through RNAi in a strain hypersensitive to exogenous RNAi.

We used RNAi to determine whether these PDAs were essential for *C. elegans* development. For each gene, we targeted regions of the gene that would permit disruption of all predicted transcripts. Co-feeding dsRNA targeting both F48E3.8 and C54G7.3 resulted in a modest delay in the development of F1 worms from N2 wild-type parents. This was evident from the presence of L3 progeny as the most advanced stage on experimental plates at 48 h in contrast to L4 progeny on matched control plates (**Figure 4A**). This effect was corroborated at 72 h when a smaller proportion of worms from experimental plates had attained adulthood in contrast to F1 adults producing eggs on control plates (**Figure 4B**). We tested whether both genes contributed to this effect, by conducting similar experiments for F48E3.8 and C54G7.3 separately using N2 wild-type worms; however, we could not detect a consistent change in development (data not shown).

To enhance the likelihood of detecting a developmental defect caused by each gene, we repeated experiments using the NL2099 strain, which carries an *rrf-3* allele rendering the strain hypersensitive to exogenous RNAi [20]. Again, we could detect no consistent effect when testing F48E3.8 alone in the *rrf-3* background (data not shown). Knockdown of C54G7.3 alone in *rrf-3* worms, though, caused a developmental delay. At 48 h, L3 progeny were the latest stage evident on control plates while L2 were the latest stage observed on experimental plates (**Figure 4C**) and at 72 h a greater proportion of F1 worms had attained adulthood in control than in experimental treatments (**Figure 4D**).

This delay in development is similar to what was observed for RNAi (by feeding) against the pharyngeal chitin synthase *chs-2* [17], where the knockdown impaired the capacity of *chs-2* to produce the new lining of the pharynx prior to each molt. Although PDA knockdown gives a less dramatic effect, the similarity with the *chs-2* data strengthens the argument that these novel PDAs may act in the *C. elegans* pharynx to modify the chitinous lining produced by *chs-2*.

None of our RNAi experiments led to a disruption in the number of intact eggs produced; eggs with normal morphology were visible on all plates. Additionally, brood sizes were normal and equivalent for all treatments (**Table 2**). Disruption of *chs-1* expression by RNAi disrupts the formation of eggshells and affects brood size [19]. Our results do not provide any evidence that these PDAs are critical to eggshell development in *C. elegans*. The integrity of these eggs and embryos is further validated by the similarity in hatching rates (measured by the presence of viable progeny at 72 hours) for matched treatments (**Table 2**).

**Table 2:** Brood Size and Progeny Viability for Reported RNAi Experiments.

		AVERAGE # OF PROGENY WITHIN 72 HRS OF PLATING P1 WORMS	
TREATMENT		Average Progeny	Progeny Viability
F48E3.8 & C54G7.3 (N2)	Control (N = 11)	40	97.7%
	Experimental (N = 7)	37	97.7%
C54G7.3(*rrf-3*)	Control (N = 9)	42	83.5%
	Experimental (N = 12)	32	88.0%

**Figure 5:**
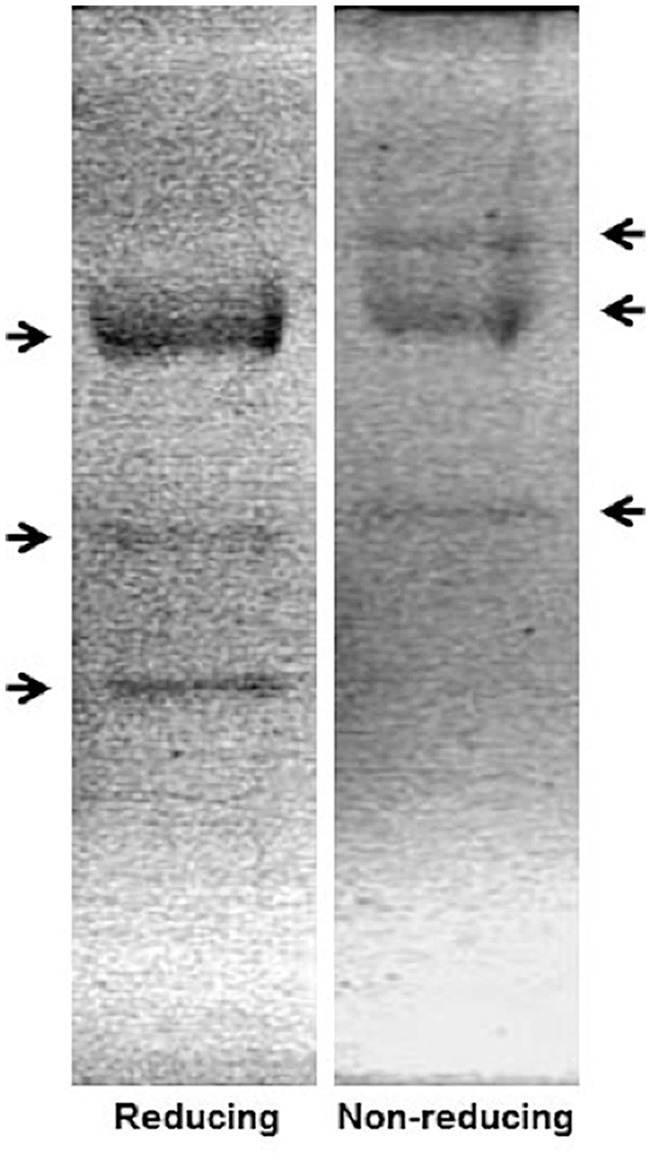
Soluble extracts of mixed-stage N2 worms deacetylate chitin *in vitro*. *C. elegans* soluble extract (14 µg total protein per lane) was denatured at 80°C and separated by SDS-PAGE under reducing and non-reducing conditions. Proteins were renatured for 24 hours at RT, enabling enzymes to act on glycol chitin embedded within the gel. Gels were stained with calcoflour. Hyperfluorescent bands mark zones where chitosan was produced. (Image is black/white reversed).

The overlap in expression patterns for these genes and the RNAi results support a role for these polysaccharide deacetylases in modifying the chitinous pharyngeal lining. We next asked whether these genes were capable of utilizing chitin as a substrate to produce chitosan. Soluble extracts of mixed stages of *C. elegans* (strain N2) deacetylated chitin *in vitro*, as demonstrated by several bands of activity in substrate gels (**Figure 5**). The hyperfluorescent bands in calcofluor-stained gels are characteristic of deacetylated regions in the glycol chitin matrix. This activity was detected in samples after separation under non-reducing or reducing conditions. Under non-reducing conditions, bands of activity appeared at 178, 165, and 132 kDa and under reducing conditions appeared at 160, 130 and 104 kDa. Comparable bands were visualized in soluble extracts from germline-ablated SS104 adult worms, although the 178 kDa band was not visualized under reducing conditions, and an additional band at 118 kDa was detected (data not shown). Hyperfluorescent bands were absent in control gels stained immediately after separation without a renaturation step, and nitrous acid-mediated depolymerization of chitosan in the gel abolished the hyperfluorescent signal as well (data not shown). This confirms the dependence of the hyperfluorescent signal on renaturation of the active enzymes and on the generation of chitosan.

In these *in vitro* experiments, the substrate is present in excess and it is noteworthy that polysaccharide deacetylases often act non-specifically on multiple substrates presented *in vitro* [21]. We attempted to quantify the extent of chitin deacetylation *in vivo* by measuring chitin and chitosan in the nematode using a differential reacetylation assay [1], [2], . Following alkali treatment, chitinase digestion of the insoluble fraction of N2 worms yielded 0.499–1.511 nmol GlcNAc per mg lyophilized worm tissue, and chitinase digestion of the equivalent fraction from germline-ablated SS104 adult worms yielded 0.050–0.170 nmol GlcNAc per mg lyophilized worm tissue (**Table 3**). Together, these results show that <10% of the chitin found in *C. elegans* is localized to somatic tissue. When mixed-stage SS104 worms grown under conditions with normal germline activity were subjected to chitinase treatment, similar yields of GlcNAc were obtained with or without prior reacetylation *in vitro* (**Table 3**). Thus any chitosan present, which would be converted to chitin by the *in vitro* acetylation, was below the level of detection. SS104 germline-ablated worms yielded product below the level of detection for the reacetylation assay (data not shown).

**Table 3:** Chitin and chitosan measured in *C. elegans* tissues.

SAMPLE & TREATMENT		GLCNAC RECOVERED FROM CHITINASE DIGESTION (NMOL/MG WORM DRY WEIGHT)
Mixed-stage worms, including adults with active germline: *measures germline and somatic chitin in various* *stages* (4 independent trials)		0.499, 0.508, 1.099, 1.511
Adult worms, germline-ablated: *measures somatic chitin in adults* (3 independent trials)		0.050, 0.055, 0.170
Mixed-stage worms, including adults with active germline	Control (mock reacetylated): *measures germline* *and somatic chitin in various stages*	0.538+/−0.061
	Reacetylated: *measures germline and somatic chitin* *and chitosan in various stages*	0.551+/−0.091

Although we could not quantify chitosan in the nematodes using this assay, our results show that small proportion of the total endogenous chitin in *C. elegans* is found in the pharynx and the vast majority is used in the chitinous eggshells. If these PDAs act as chitin deacetylases *in vivo*, they target a small proportion of the total endogenous chitin in fertile hermaphrodites.

**Figure 6:**
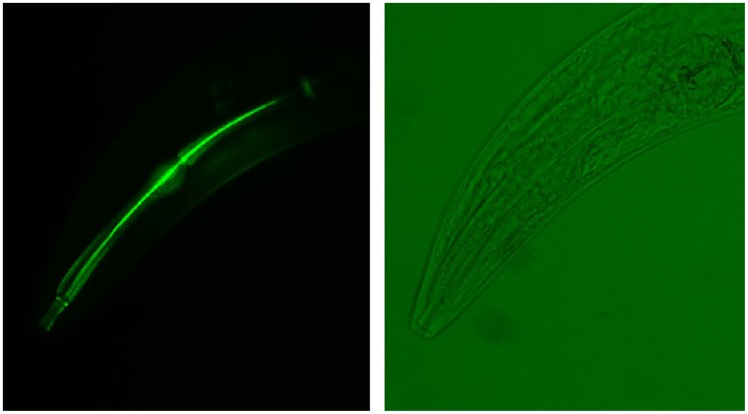
Eosin Y, a dye specific for chitosan in the fungal cell wall, stains the *C. elegans* pharynx. N2 worms were stained without fixation using Eosin Y and visualized with an Olympus BX40 epifluorescent microscope with a Chroma 31001 filter (left) or with bright field (right).

Eosin Y has previously been reported to stain chitosan specifically in the fungal cell wall of unfixed *C. neoformans* [3]. We stained living, unfixed worms with this dye and noted a highly reproducible staining apparent in the lumen of the buccal cavity, procorpus, metacorpus and isthmus, and the flaps of the terminal bulb (**Figure 6**). This staining occurs in various developmental stages and overlaps with the pattern seen in fixed worms stained for chitin [19]. Solutions of a non-specific fluorescent dye, 4-methlyumbelliferone (4-MU), were used in the same staining protocol to test whether ingestion or trapping of dye, rather than specific staining, would generate an equivalent fluorescent pattern in the unfixed worms. No such luminal staining was detected in worms treated with 4-MU under matching conditions.

## Discussion

We have conducted the first functional analysis of polysaccharide deacetylases in nematodes, starting with the genes F48E3.8 and C54G7.3 in *C. elegans*. The somatic expression of PDA domain-encoding transcripts of F48E3.8 and C54G7.3 in *C. elegans*, demonstrated by our RT-PCR, corroborates the pharyngeal expression of these genes described by Gaudet and Mango (2002) and observed in publicly available *in situ* hybridization data sets for the genes (NEXTDB, http://nematode.lab.nig.ac.jp/). Our results demonstrate that somatic tissues contain gene products capable of deacetylating chitin when presented with the substrate *in vitro*.

Genes encoding PDAs are widely distributed in the *Nematoda* as we have identified homologs in representatives from all clades except Clade IVa, where sequence availability may be a limiting factor (**Table 1**). In *C. elegans*, F48E3.8 and C54G7.3 are the two representatives of the polysaccharide deacetylase family. These are large genes of ∼9.7 kb and 13.3 kb, respectively. In the closely related *C. briggsae*, we have identified CBG14800 as the ortholog of *Ce-*C54G7.3 gene. CBG14800 (which encodes a predicted PDA domain) and CBG14801 (which encodes a predicted CBD domain) are annotated as separate but adjacent genes in *C. briggsae*; however, they likely represent one chitin deacetylase-encoding gene that is similar to *Ce*-C54G7.3. In C54G7.3, predicted exons 11 and 12 are separated by an unusually large ∼4 kb intron. CBG14801 aligns with *Ce*-C54G7.3 predicted exons 1–11; CBG14800 aligns with *Ce*-C54G7.3 predicted exons 12–33. Clones generated from *C. elegans* cDNA include transcripts spanning this large intron (Wormbase 229) confirming that these units represent a single gene that includes a large intron in *C. elegans*. Using RT-PCR, we have confirmed the existence of transcripts spanning this intron (data not shown). The small size of the other C54G7.3 orthologs, *C. brenneri* CBN-LGX-1 and *C. remanei* CRE_00232 (**Table 1**), suggests that the annotation of this locus as two separate genes has also been repeated during transcript prediction in these other species, and should be revisited.

Two PDA-encoding homologs have been identified in all the *Caenorhabdita* (**Table 1** and **Figure 1**) suggesting that a gene duplication event occurred following the separation of the genus from other nematodes and prior to the speciation leading to four separate species. The relative proximity of the two genes on the × chromosome in *C. elegans* supports the argument that paralogs in each of the *Caenorhabdita* may have resulted from a relatively recent duplication event within this genus. The high level of sequence similarity among *Ce*-C54G7.3 and its orthologs (**Figure 2**) suggests the possibility of a higher level of functional constraint in residues among these proteins when compared to *Ce*-F48E3.8 and its orthologs.

Luschnig *et al.* (2006) and Wang *et al.* (2006) first elucidated a role for chitin deacetylases in invertebrates. They demonstrated that the Serp and Verm proteins of *Drosophila* act on the chitinous lining of the embryonic dorsal trunk to regulate longitudinal extension of this tracheal tube, following the deposition of luminal chitin by the chitin synthase Kkv/CS-1. Although more complex in form than the *Drosophila* dorsal trunk, the nematode pharynx also contains a tubular structure lined by epithelial cells from which chitin synthase secretes a luminal chitin lining. The wide distribution of PDAs in nematodes suggests that they may be involved in fundamental, highly conserved processes that are key to roundworm development. We propose a model wherein nematode PDAs act on the pharyngeal lining, consequently affecting development in roundworms. Deacetylation converts chitin, an inert, uncharged homopolymer, to chitosan, a partially charged molecule, with different fibrillar structure. In the *C. elegans* pharynx, this transition could alter the proximity of the pharyngeal lining to the underlying epithelium as changes in charge or hydration cause conformational change. The creation of free amine groups by deacetylation may also allow new covalent linkages to be formed. This raises the prospect of signaling between extracellular chitosan and underlying cells through covalent linkages between chitosan and proximal proteins (integral or peripheral membrane proteins or other proteins of the ECM). Such signaling could control growth of the pharynx, either during organogenesis or in the intermolt periods. The chitinous lining of the pharynx is re-synthesized by CHS-2 prior to each molt [17], [18], [19]; but unlike insects, nematodes undergo considerable growth between molts, and the pharynx lengthens as well. Deacetylation of the pharyngeal lining may directly regulate radial or longitudinal growth, through physical and chemical changes that alter the interaction between this extracellular lining and the underlying myoepithelium. Deacetylation may be an important mechanism for coordinating growth between pharyngeal cells and their protective barrier.

Regulating pharyngeal growth is a particularly complex problem in organisms like the filarial parasites, where adult worms may be many centimeters long and the pharynx consequently undergoes significant longitudinal extension following the final molt. A stage specific proteome for *B. malayi* has recently been published [22] and the results demonstrate that the homolog Bml_33340, which we have identified (**Table 1**), has a protein product present in stages ranging from microfilaria (equivalent to the *C. elegans* L1) through to adult male and female worms. Bml_33340 is a somatic PDA and it remains possible that this protein may act to regulate pharyngeal development in *B. malayi*.

Our results show that PDAs affect nematode development and we propose that this results from the action of the PDAs on the chitin lining the pharynx. However, deacetylation of pharyngeal chitin may have other implications for nematodes, and indeed, PDAs may have alternative endogenous or exogenous targets in various free-living or parasitic nematodes (**Table 4**).

**Table 4:** Potential roles for nematode PDAs.

TARGET		EFFECT	OUTCOME
**Endogenous**	**Pharyngeal chitin**	Facilitates signaling to underlying epithelium;control of length/diameter of chitinous lining	Pharyngeal morphogenesis
		Affects recognition of the pharyngeal liningby other enzymes	Protection of gut from exogenous chitinases
	**Eggshell chitin**	Alters physical properties of the macromolecule	Hatching or eggshell remodeling
	**GAGS or other acetylated hexosamines**	Affects various context-specific developmental functions	Morphogenesis
**Exogenous**	**Xylan (Plant hosts)**	Permits penetration of host tissues	Virulence
	**GAGs (Vertebrate hosts)**	Permits penetration of host tissues	Virulence

For example, pharyngeal PDAs may serve to protect the chitinous lining from degradation by chitinases. In *S. pneumoniae*, the peptidoglycan deacetylase *Sp*PgDA is a virulence factor that deacetylates peptidoglycan in the cell wall, protecting the bacterium from degradation by lysozymes [14]. Pharyngeal PDAs could serve an analogous role in parasitic nematodes by protecting the pharyngeal lining from degradation by chitinases produced as part of the innate immune response of plants and vertebrates. Similarly, free-living nematodes may utilize this deacetylation of pharyngeal chitin to confer protection from bacterial and fungal chitinases produced by environmental microbes.

Other acetylated polysaccharides may be targets for the nematode PDAs we have identified. Caufrier et al. (2003) showed that members of this enzyme family may act on various N- and O-acetylated substrates. Heparan sulfate, a glycosaminoglycan (GAG) is required for *C. elegans* pharyngeal morphogenesis [23] and members of the PDA family of enzymes have been shown to deacetylate the GAGs heparan sulfate and heparin [11]. Just as endogenous GAGs could be targets of these deacetylases, so too might GAGs in vertebrate host tissues be targets for parasite enzymes, facilitating infection.

Plant parasitic nematodes invade tissues fortified by various carbohydrates including xylan (a complex variety of polysaccharides including the pentose sugar xylose). Members of the PDA family may act to weaken targeted plant tissue, facilitating invasion by plant parasites. Intriguingly, the homolog we have identified from the plant parasitic nematode *Meloidogyne incognita* (Msp9) is likely produced and secreted from esophageal gland cells, since it bears a signal peptide and is expressed in these cells [24]. The function of this enzyme has not been elucidated, but it is presumed to function as a plant virulence factor like other proteins secreted from the gland cells.

We have demonstrated the expression of both *C. elegans* genes, F48E3.8 and C54G7.3, in the soma, but we cannot exclude the possibility that the genes may also function in the germline, as eggshell chitin represents roughly 90% of all chitin synthesized in the nematode. We were unable to confirm significant levels of chitosan in fertile hermaphrodites, but cannot rule out a role for PDAs in eggshell development. Among the filarial parasites, certain species (including *Brugia spp.*) exhibit a remodeled eggshell, or sheath, surrounding their first stage larvae. Two PDAs were identified in *B. malayi*, including one encoded in the genome of the *Wolbachia* endosymbiont known to be essential to the nematode’s fecundity. WolBm0147 is a *bona fide* product found in the *Wolbachia* proteome [22] and it is an intriguing prospect that the WolBm0147 protein (which has a signal peptide) may participate in remodeling the eggshell.

Using a differential reacetylation assay, we were unable to biochemically identify chitosan in *C. elegans* (with or without the abundant chitin present from the germline). Two possibilities may explain our results: first, chitosan may be produced in the worms then coupled to some other molecule rendering the amino group inaccessible for *in vitro* reacetylation and chitinase degradation in our experiments; alternatively, the level of chitosan present may be below the level of detection afforded by a colorimetric assay. In the protist *E. invadens*, Das et al. (2006) have detected approximately 60 nmol GlcNAc released per mg cyst (wet weight), attributing roughly 15 nmol to chitin and 45 nmol to chitosan. These quantities are 40-fold greater than the levels of the carbohydrate we observe in wild-type *C. elegans* and 400-fold greater than the levels when compared to germline-deficient worms; given that our values are normalized to lyophilized weight, it suggests that the difference in levels of the carbohydrate per unit mass are far greater than this direct comparison. Our inability to detect chitosan by this method, then, may simply reflect the low levels of the carbohydrate present in the pharynx.

Nematodes may utilize PDAs to target endogenous chitin and GAGS or exogenous substrates, thereby influencing morphogenesis, immunity and virulence. Our results suggest that disrupting the function of polysaccharide deacetylases disrupts nematode development irrespective of their target. Importantly, we have found no close homologs in human sequence, suggesting that polysaccharide deacetylases (including chitin deacetylases) may prove to be a valid target for the development of interventions that selectively affect human parasites.

## Materials and Methods

### Strains

The wild-type *C. elegans* Bristol N2 strain, the SS104 strain (carrying the temperature-sensitive *glp-4* allele, bn2), and the NL2099 strain (carrying the *rrf-*3 allele, pk1426) were used for these experiments.

### Worm Growth and Sampling

Worms for developmental time course experiments were grown on solid media at 25°C with sampling performed as previously described [17], [25], after the following minimum numbers of arrested L1’s were plated: 25,000 worm/plate for plates to be sampled for L1’s and L2’s; 12,500 worms/plate for plates to be sampled for L3’s and L4’s; 6250 worms/plate for plates sampled to gather adults. (A larger number of worms were used for time courses with the SS104 strain to account for the absence of a germline.) Under our conditions, 75% of all hermaphrodites had identifiable vulvas by 72 h and 100% had identifiable vulvas by 96 h. At specific time points, worms were harvested by washing plates using ice-cold 0.1 M NaCl, separated from bacteria by flotation on 30% sucrose, suspended in 1 mL Trizol reagent, and immediately frozen at −80°C.

Worms for enzyme activity assays were grown in liquid culture using complete S-Basal [26] and collected as above. 5 mL aliquots of the wet worm pellets were stored with 1 µM leupeptin, 0.2 µM pepstatin, 1 µg/mL aprotinin and 500 µM PMSF at −20°C. Thawed worm samples were extracted by sonification and cleared by centrifugation for 15 min at 12,500×g.

Worms used for chitin/chitosan quantification were similarly grown and purified from liquid culture. 1.25 mL-1.50 mL wet worm pellets were stored in 2.0-mL screw cap tubes (with no protease inhibitors) at −20°C. Samples were lyophilized for ∼48 hours before dry mass was determined.

### Identification and Phylogenetic Analysis of Nematode CDAs

We used the predicted or confirmed catalytic domain peptide sequence of *Ce-*C54G7.3 and *Sp*PDA (a peptidoglycan deacetylase characterized from the bacterium *Streptococcus pneumonia*) to search for additional genes with predicted polysaccharide deacetylase domains in *C. elegans*, the parasitic nematode *Brugia malayi*, and the Brugian endosymbiotic bacterium, *Wolbachia sp*. C54G7.3. Three newly identified PDA-encoding sequences from *C. elegans* (F48E3.8) and *B. malayi* (Bml_33340 and WolBm0147) were then used to search for additional nematode homologs. For all searches we used a combination of BLAST and PSI-BLAST to mine the NCBI non-redundant sequences for the phylum *Nematoda* as a whole, for select nematodes, and (where relevant) for the endosymbiont *Wolbachia*. Additional searches were conducted against Sanger Institute genome sequencing data for *Onchocerca volvulus* (http://www.sanger.ac.uk/cgi-bin/blast/submitblast/o_volvulus) and for other species accessible through http://www.nematode.net. We also searched for homologs in humans. (The accession numbers for all protein sequences as well as the specific region of each used for alignments are provided in **Table 1** and **Table S1**.).

The predicted or confirmed catalytic domain peptide sequences for the nematode homologs were used to create an alignment using ClustalW, which included sequences from bacteria (*S. pneumonia* peptidoglycan deacetylase), fungi *(C. neoformans* chitin deacetylases), and insects (*D. melanogaster* and *T. castaneum* chitin deacetylases). The alignment was created with the following parameters: pairwise alignment was generated using a gap opening penalty of 10 and gap extension penalty of 0.1, while multiple alignment was generated using a gap opening penalty of 3.0 and gap extension penalty of 1.8. This alignment was used as the basis for creating a minimum evolution tree in Mega4.0 [27].

### RT-PCR

RNA was isolated from worms by homogenization in Trizol reagent (Invitrogen), treated with Turbo DNAse (Ambion) and quantified using a NanoDrop 2000 Spectrophotometer (Thermo Scientific). We diluted samples to 0.2 µg/µL and performed cDNA synthesis for each time point using the Protoscript M-MuLV First Strand cDNA Synthesis Kit (New England BioLabs) using 2 µg of RNA (+/− RT) in a 40 µL reaction.

PCR amplifications used 5 µL of each cDNA template in a 50 µL reaction along with 0.8 mM dNTP mix (USB), 1 µM of each primer (Operon), and Taq with ThermoPol Buffer (NEB). All primers and PCR conditions are listed in **Table S2**.

### RNAi

In order to generate dsRNA targeting *lgx-1*/C54G7.3, a large fragment of the gene from the yk1621b03 clone (kindly provided by Dr. Yuji Kohara) was subcloned to the v28i Litmus Vector (NEB) using EcoRI and XbaI sites. In order to generate dsRNA targeting the F48E3.8 gene, a 0.9-kb fragment of the yk1130a03 clone (kindly provided by Dr. Yuji Kohara) was subcloned to the v28i Litmus Vector (NEB) using EcoRI and PstI sites. RNAi experiments utilized the feeding protocol previously described [28]. *E. coli* (HT115[DE3]) expressing dsRNA targeting one or both PDA genes were fed to individual L3 worms. The parent was removed from each plate at 48 h. The F1 generation of each was then screened at 48 h and again at 72 h using blind scoring. Control experiments used the v28i vector with no insert.

### Enzyme Activity Assays

CDA activity was visualized using the substrate gel method as described by Trudel and Asselin (1990). SDS-PAGE was used to separate soluble worm extracts (14 µg per lane) under reducing or non-reducing conditions in gels containing 0.01% glycol chitin. In order to visualize CDA activity following electrophoresis, the gels were incubated for 24 h at room temperature in a renaturation solution containing 1% Triton X-100 in 50 mM Hepes-KOH (pH 7.0), on a shaker. The gels were then stained with 0.01% Calcofluor in 0.5 M Tris-HCl (pH 9) solution for approximately 2 hours, followed by constant washes of picopure water. Deacetylated chitin generates hyperfluorescent bands over the background staining of chitin under UV illumination [29]. Replicate gels developed by staining without renaturation were used as negative controls to demonstrate dependence of the signal on enzymatic digestion.

### Chitin and Chitosan Detection

The chitin and chitosan content from lyophilized worm pellets was quantified using *in vitro* reacetylation, chitinase degradation and a Morgan-Elson assay modified for a 96-well plate format as previously described [1], [30].

For eosin Y staining, mixed stage N2 worms were washed and resuspended in 500 µL citrate-phosphate buffer, pH 6.0 (0.2 M NaH_2_PO_4_ and 0.1 M KCitrate) before 15 µL of eosin Y stock (5 mg/ml in 70% ethanol) was added. Tubes were incubated at RT in the dark for 10 min, then spun for 5 min at 600×g to wash. The supernatant was replaced with fresh citrate-phosphate buffer, and this wash step was repeated for a total of 5 washes. The samples were observed and imaged using an Olympus BX40 epifluorescent microscope with a Chroma 31001 filter. Non-specific staining through ingestion of fluorescent dye was tested using 4-methylumbelliferone at 0.15 mg/mL as a control.

## Supporting Information

Table S1 **Additional Non-Nematode Sequences Used in Protein Alignments and Phylogenetic Analysis.**
(DOC)

Table S2 **Primers and PCR Conditions Used for Amplification.**
(DOC)

## References

[1] Das S, Van Dellen K, Bulik D, Magnelli P, Cui J (2006). The cyst wall of Entamoeba invadens contains chitosan (deacetylated chitin).. Mol Biochem Parasitol.

[2] Banks IR, Specht CA, Donlin MJ, Gerik KJ, Levitz SM (2005). A chitin synthase and its regulator protein are critical for chitosan production and growth of the fungal pathogen Cryptococcus neoformans.. Eukaryot Cell.

[3] Baker LG, Specht CA, Donlin MJ, Lodge JK (2007). Chitosan, the deacetylated form of chitin, is necessary for cell wall integrity in Cryptococcus neoformans.. Eukaryot Cell.

[4] Arakane Y, Dixit R, Begum K, Park Y, Specht CA (2009). Analysis of functions of the chitin deacetylase gene family in Tribolium castaneum.. Insect Biochem Mol Biol.

[5] Luschnig S, Batz T, Armbruster K, Krasnow MA (2006). serpentine and vermiform encode matrix proteins with chitin binding and deacetylation domains that limit tracheal tube length in Drosophila.. Curr Biol.

[6] Wang S, Jayaram SA, Hemphala J, Senti KA, Tsarouhas V (2006). Septate-junction-dependent luminal deposition of chitin deacetylases restricts tube elongation in the Drosophila trachea.. Curr Biol.

[7] Dixit R, Arakane Y, Specht CA, Richard C, Kramer KJ (2008). Domain organization and phylogenetic analysis of proteins from the chitin deacetylase gene family of Tribolium castaneum and three other species of insects.. Insect Biochem Mol Biol.

[8] John M, Rohrig H, Schmidt J, Wieneke U, Schell J (1993). Rhizobium NodB protein involved in nodulation signal synthesis is a chitooligosaccharide deacetylase.. Proc Natl Acad Sci U S A.

[9] Gaudet J, Mango SE (2002). Regulation of organogenesis by the Caenorhabditis elegans FoxA protein PHA-4.. Science.

[10] Guo W, Li G, Pang Y, Wang P (2005). A novel chitin-binding protein identified from the peritrophic membrane of the cabbage looper, Trichoplusia ni.. Insect Biochem Mol Biol.

[11] Berninsone P, Hirschberg CB (1998). Heparan sulfate/heparin N-deacetylase/N-sulfotransferase. The N-sulfotransferase activity domain is at the carboxyl half of the holoenzyme.. J Biol Chem.

[12] Blaxter ML, De Ley P, Garey JR, Liu LX, Scheldeman P (1998). A molecular evolutionary framework for the phylum Nematoda.. Nature.

[13] Blair DE, van Aalten DM (2004). Structures of Bacillus subtilis PdaA, a family 4 carbohydrate esterase, and a complex with N-acetyl-glucosamine.. FEBS Lett.

[14] Blair DE, Schuttelkopf AW, MacRae JI, van Aalten DM (2005). Structure and metal-dependent mechanism of peptidoglycan deacetylase, a streptococcal virulence factor.. Proc Natl Acad Sci U S A.

[15] Blair DE, Hekmat O, Schuttelkopf AW, Shrestha B, Tokuyasu K (2006). Structure and mechanism of chitin deacetylase from the fungal pathogen Colletotrichum lindemuthianum.. Biochemistry.

[16] Thein MC, McCormack G, Winter AD, Johnstone IL, Shoemaker CB (2003). Caenorhabditis elegans exoskeleton collagen COL-19: an adult-specific marker for collagen modification and assembly, and the analysis of organismal morphology.. Dev Dyn.

[17] Veronico P, Gray LJ, Jones JT, Bazzicalupo P, Arbucci S (2001). Nematode chitin synthases: gene structure, expression and function in Caenorhabditis elegans and the plant parasitic nematode Meloidogyne artiellia.. Mol Genet Genomics.

[18] Fanelli E, Di Vito M, Jones JT, De Giorgi C (2005). Analysis of chitin synthase function in a plant parasitic nematode, Meloidogyne artiellia, using RNAi.. Gene.

[19] Zhang Y, Foster JM, Nelson LS, Ma D, Carlow CK (2005). The chitin synthase genes chs-1 and chs-2 are essential for C. elegans development and responsible for chitin deposition in the eggshell and pharynx, respectively.. Dev Biol.

[20] Simmer F, Tijsterman M, Parrish S, Koushika SP, Nonet ML (2002). Loss of the putative RNA-directed RNA polymerase RRF-3 makes C. elegans hypersensitive to RNAi.. Curr Biol.

[21] Caufrier F, Martinou A, Dupont C, Bouriotis V (2003). Carbohydrate esterase family 4 enzymes: substrate specificity.. Carbohydr Res.

[22] Bennuru S, Meng Z, Ribeiro JM, Semnani RT, Ghedin E (2011). Stage-specific proteomic expression patterns of the human filarial parasite Brugia malayi and its endosymbiont Wolbachia.. Proc Natl Acad Sci U S A.

[23] Franks DM, Izumikawa T, Kitagawa H, Sugahara K, Okkema PG (2006). C. elegans pharyngeal morphogenesis requires both de novo synthesis of pyrimidines and synthesis of heparan sulfate proteoglycans.. Dev Biol.

[24] Huang G, Gao B, Maier T, Allen R, Davis EL (2003). A profile of putative parasitism genes expressed in the esophageal gland cells of the root-knot nematode Meloidogyne incognita.. Mol Plant Microbe Interact.

[25] Johnstone IL, Barry JD (1996). Temporal reiteration of a precise gene expression pattern during nematode development.. EMBO J.

[26] Wood WB, editor (1988). The Nematode Caenorhabditis elegans.. Cold Spring Harbor, New York: Cold Spring Harbor Laboratory Press.

[27] Kumar S, Tamura K, Nei M (2004). MEGA3: Integrated software for Molecular Evolutionary Genetics Analysis and sequence alignment.. Brief Bioinform.

[28] Kamath RS, Ahringer J (2003). Genome-wide RNAi screening in Caenorhabditis elegans.. Methods.

[29] Trudel J, Asselin A (1990). Detection of chitin deacetylase activity after polyacrylamide gel electrophoresis.. Anal Biochem.

[30] Bulik DA, Olczak M, Lucero HA, Osmond BC, Robbins PW (2003). Chitin synthesis in Saccharomyces cerevisiae in response to supplementation of growth medium with glucosamine and cell wall stress.. Eukaryot Cell.

