# Supplementary material for: Pharyngeal Polysaccharide Deacetylases Affect Development in the Nematode C. elegans and Deacetylate Chitin In Vitro
Source: PLoS One. 2012 Jul 13;7(7):e40426. doi: 10.1371/journal.pone.0040426 (PMC3396651; doi:10.1371/journal.pone.0040426)
Supplement: Table S1 — Additional Non-Nematode Sequences Used in Protein Alignments and Phylogenetic Analysis. (DOC) [file pone.0040426.s001.doc]

**Table S1. Additional Non-Nematode Sequences Used in Protein Alignments and Phylogenetic Analysis**

| **PHYLA** | **SPECIES** | **NCBI ACCESSION NUMBER** | **PREVIOUSLY ASSIGNED NAMES** | **TOTAL # OF RESIDUES IN ARCHIVED SEQUENCE** | **RESIDUES INCLUDED IN ALIGNMENTS AND TREE RELATIVE TO ARCHIVED SEQUENCE** |
| --- | --- | --- | --- | --- | --- |
| Bacteria | *Streptococcus pneumoniae* | 2C1G_A | SpPGDA | 431 | 235-390 |
| Protista | *Entamoeba histolytica* | XM_651817 | EhCDA1 | 275 | 53-211 |
|  |  | XM_651264 | EhCDA2 | 262 | 57-223 |
|  | *Entamoeba invadens* | DQ284499 | EiCDA1 | 281 | 54-211 |
|  |  | DQ284500 | EiCDA2 | 262 | 57-223 |
|  |  | DQ284501 | EiCDA3 | 293 | 84-249 |
| Fungi | *Cryptococcus neoformans* | XP_571516 | CDA1 | 470 | 158-336 |
|  |  | XP_570561 | CDA2 | 458 | 157-327 |
|  |  | XP_571200 | CDA3 | 410 | 123-294 |
|  |  | XP_568540 | FPD1 | 249 | 38-200 |
| Insecta | *Drosophila melanogaster* | NP_730444 | DmCDA1, SERP | 541 | 200-359 |
|  |  | NP_730443 | DmCDA2, VERM (Isoform B) | 549 | 207-367 |
|  |  | NP_609806 | DmCDA3, DmChLD3 | 577 | 243-405 |
|  | *Tribolium castaneum* | ABU25223 | TcCDA1 | 534 | 192-352 |
|  |  | ABU25224 | TcCDA2 (Isoform A) | 535 | 191-351 |
|  |  | ABW74145 | TcCDA3 | 505 | 180-336 |
|  |  | ABW74146 | TcCDA4 | 490 | 142-297 |
|  |  | ABW74147 | TcCDA5 (Isoform A) | 1131 | 800-954 |
|  |  | ABW74149 | TcCDA6 | 403 | 52-209 |
|  |  | ABW74150 | TcCDA7 | 374 | 54-211 |
|  |  | ABW74151 | TcCDA8 | 376 | 54-211 |
|  |  | ABW74152 | TcCDA9 | 381 | 54-212 |
