# Supplementary material for: Pharyngeal Polysaccharide Deacetylases Affect Development in the Nematode C. elegans and Deacetylate Chitin In Vitro
Source: PLoS One. 2012 Jul 13;7(7):e40426. doi: 10.1371/journal.pone.0040426 (PMC3396651; doi:10.1371/journal.pone.0040426)
Supplement: Table S2 — Primers and PCR Conditions Used for Amplification. (DOC) [file pone.0040426.s002.doc]

Table S2. Primers and PCR Conditions Used for Amplification

| **TARGET** | **PRIMERS** | **PCR CONDITIONS** |
| --- | --- | --- |
|  | (UNDERLINED RESIDUES ANNEAL WITH TARGET SEQUENCES) |  |
| F48E3.8PDA | CCTTCAAGACTTCTGACCGAATGCCCG (F) GTTTGTGGCCAATATGGGCTGCTGACC (R) | 94°C, 3 minutes; [94°C, 1 minute; 58.0°C, 1 minute; 72°C, 3 minutes]x30; 72°C, 10 minutes |
| C54G7.3PDA | AAGACGAGCTCCGGGTGTCTCCGC (F) CGCTAGAATTCTCCTCTTCAGGAGCTCCACCGAAC (R) | 94°C, 3 minutes; [94°C, 1 minute; 61.4°C, 1 minute; 72°C, 3 minutes]x2; [94°C, 1 minute; 64.7°C, 1 minute; 72°C, 3 minutes]x28; 72°C, 10 minutes |
| *ama-1* (F36A4.7) | TTCCAAGCGCCGCTGCGCATTGTCTC (F) CAGAATTTCCAGCACTCGAGGAGCGGA (R) | 94°C, 3 minutes; [94°C, 1 minute; 60.0°C, 1 minute; 72°C, 3 minutes]x30; 72°C, 10 minutes |
| *chs-1* (T25G3.2) | TCTTGGATCCTCTCGCCAGATGCCTTGTTCTCAG (F) ACAAGAATTCGCCAAGAAGCGTGAAATCCCTCGC (R) | 94°C, 3 minutes; [94°C, 1 minute; 59.9°C, 1 minute; 72°C, 3 minutes]x2; [94°C, 1 minute; 63.7°C, 1 minute; 72°C, 3 minutes]x28; 72°C, 10 minutes |
| *chs-2* (F48A11.1) | ACAAGGATTCACCATGTTCCGAAACGTGAAACGC (F) ACAAGAATTCAAGCAGCAGACAACGTAGATGCAG (R) | 94°C, 3 minutes; [94°C, 1 minute; 58.5°C, 1 minute; 72°C, 3 minutes]x2; [94°C, 1 minute; 62.0°C, 1 minute; 72°C, 3 minutes]x28; 72°C, 10 minutes |
| c*ol-19* (ZK1193.1) | TTGGATCCTGCGGAGTACTTGTGTGCG (F) CGGCATCCTCTCCCTTTTGTCCTGG (R) | 94°C, 3 minutes; [94°C, 1 minute; 61.1°C, 1 minute; 72°C, 3 minutes] x30; 72°C, 10 minutes |
